# Causes Retarding Dental Progress

**Published:** 1863-08

**Authors:** Wm. H. Atkinson


### DENTAL REGISTER OF THE WEST.

VOL. XVII.] AUGUST, 1863. [NO. 8.

### Original Essays and Communications.

### CAUSES RETARDING DENTAL PROGRESS.

(Read before the American Dental Convention, Aug. 8, 1863.)

BY WM. H. ATKINSON, M. D.

Dental progress, like all other forms of reformatory or advancing movement, is retarded by all the causes and any causes that lay the foundation for its necessity. In general terms, we may safely aver that *ignorance* of and failure to obey *law* is the prime cause for the necessity of dental science, so far as it pertains to practical results to be attained by the teaching and practice of dental science and manipulative art. There is a striking resemblance between complete states of ignorance (or lack of knowledge) and knowledge in all the practical applications of the purposes of the mind. The old homely phrase, "he who *knows* nothing *fears* nothing," might as truly be coupled with "he who *knows* all things is superior to all influences of *doubt*, and is therefore above *fear* of every variety."

It is the nature of mind spontaneously to essay to execute its purposes; and hence it acts upon the supposition that it is omnipotent until by failure it discovers the limit of its *present* effective range. It is only the timid, half made-up sort of mind that instinctively entertains a doubt of accomplishing the spontaneity of its desires and deigns to debate probabilities. The confident, well-made-up sort of sentient nature will scarcely entertain a doubt of its ability to perform its behests in the face of many failures to effect that end. Not having "calculated the chances," as ripe experience has taught the mutilated and fatigued specimens of this sort of mind to do in subsequent pioneerings, it is difficult for them to accept the unpleasant truth that it requires time and repeated effort to enable them to practicalize the purpose that is so easily and clearly formed without conscious effort on their part. The will to do is present; but how to perform they find not at once.

Just in the degree that we become masters theoretically and accomplished workmen practically will we be confident of success in our efforts to accomplish that which we undertake. But if we should miscalculate the principles of the case, or our skill to perform the work of redemption, failure will open our vision to see our lack of knowledge or command of our powers, and thus we are prepared for intelligent efforts at further attempts to accomplish the work, having learned the way by which it can not be done. The failure having resulted from a stretching too far beyond the measure of power of diagnosis or performance, should not discourage us from further attempt to accomplish new and untried feats. But it should admonish us to look well to the principles involved in every case, new or trite, that we may keep within due bounds, and progress in steps where tropes would too distantly connect the ideal and the practical.

Short pupilage implies imperfect instruction, and imperfect instruction lays the foundation of all failures to accomplish desirable results. Does any deny this, and point to many of those in the first ranks of our honorable body, and promise that they were either pupils for very limited terms, or actually never acknowledged any one as tutor at all? My reply is, that this does not militate against the position assumed. For the closest scrutiny will prove that undying effort, study, and labor has had much more to do with the making of accomplished dentists than the most brilliant genius.

To be sure, the pupilage and preparation may not have been attained in any dental office or school especially devoted to that science; but the education of the eye, hand, head, and heart must have been attained *before* the performance of any superior work pertaining to any department of dental art.

But all the brilliant success of this class of our members will not warrant us in assuming that genius and not tutorage and pupilage is the pre-requisite for a useful and successful dentist. Take the testimony of the whole career of any one who has been eminently successful, and my word for it he was a working man, and also had suffered immensely from failures to realize in practical results the good desires which prompted him to enter an untrodden field for the good to be accomplished. Those who stand highest among us are indebted to a medical education in the schools of anatomy, chemistry, and therapeutics, etc., or to a long line of inflictions of needless misery, suffering, and loss of teeth and comfort to numerous patients, by which they have been educated up to their present ability. And who in his senses would knowingly choose the latter?

Probably the very greatest cause of the retardation of progress is, that we are too apt at looking for the easiest way of performing our work, instead of that way of effecting it that will bring the greatest good to those about us, without bringing everlastingly into the foreground of consideration, "but what is the effect upon *me* going to be?"

A poor, foolish, selfish fly has for the tenth time flown dash against a mirror at my back, in which the representative of an open window is very plainly delineated. Sometimes he comes with all his force against the thick, hard, polished glass with a thump and a rebound that stuns him for a moment; but recovering his equilibrium, he circles once or twice within the space of a few feet, and dash away he goes again to butt his poor head against the unyielding smooth reflector; and so he repeats and repeats the effort to go through the glass to the, to him, inviting field beyond, never stopping to consider that he may be wrong, until by utter exhaustion he at last falls bewildered, fatigued, and overcome to the soft reception of the delicate Brussels beneath, where he, as it were by chance, finds himself facing not the image of the window, but the window itself. As soon as he has somewhat recovered, he rises with the vigor and confidence of his former efforts, and lo! he scales the open vista, and to his great joy finds himself in the freedom of the open air, with liberty to soar to his heart's content, till he becomes food for some member of the caprimulgus family; his incorporation into whose body we will not now attempt to trace.

I fear we too often retard our own progress as did the poor fly, by not being instructed by our failures and the rebuffs we meet. The fly acted upon the "try, try again" doctrine, and it is a doubtful matter whether he was ever aware of the reason of his success in the end. And unless we vary our plans and procedures we will be like him unsuccessful, unless by a fortuity our attention is directed to another line of tactics.

Failures *well observed* are quite as sure to advance us as unexpected successes; for failures sting us into closer investigations which point out the reason of non-success, while spontaneous successes lull us to a soporific confidence in our "*genius*," which is the more common cause of our attempting things too far ahead of our development for regular and certain accomplishment, and induces us to depend upon our "good luck" and boasted "*habit* of succeeding."

The next most potent cause retarding dental progress, is the low standard at which we aim. Most dentists, as most men, are quite satisfied to be "as good as their neighbor," while he never becomes great or truly progressive who has not deeply burned into his every purpose and every act the desire to excel not only his immediate neighbors and associates, but his own best former effort. As the mind as well as the body becomes strengthened in the directions most used, so the "impudence of hope" habitually indulged finally becomes the medium of inspirations, the consciousness of which makes him who exercises it walk the earth an anthropos indeed.

We need not fear that we shall get too far ahead in doing practical good to be regarded as of the main body of the profession. For such is the genius of our body that the more they become convinced that they are behind, the more will they bestir themselves to prove that they are as well endowed and as necessary to the body politic "as any other man." And thus the ambition of laudable emulation will give place to the hollow practice of sheer pretense, or drive the unworthy from the vineyard as cumberers of the ground, which will no longer accept obsolete mutilations for preservations and restorations of deranged portions thereof.

Being satisfied with mediocrity prevents or limits ambition, and lack of this quality stands in the way of the desire to associate; and absence of social intercourse deprives us of a just knowledge of the true standing of our fellow-practitioners; so that when we by accident do hear of some great advance having been attained in some department of our specialty, we are slow to believe it, because it is so much above our accustomed state of mind that we are apt to regard it as impossible, or at most quite improbable that any mind has so far outstripped our plodding state.

If the past is any criterion by which to judge of the future, we may confidently look for retardation to our progress in the exact ratio that we isolate ourselves and ignore each other's improvements and ideas of advancement, or we may be sure of rapid progress in the degree in which we recognize each other, fraternally associate, support the dental journals, and, above all, the dental schools.

This last is the great sheet-anchor to all correct dental physiology, pathology, and mode of practice. The schools are necessarily the focalizing points of the highest attainments, and the broadest fields of clinical application of doctrines and methods.

A great sin lays at the door of most of our best operators in this respect. Having a good paying practice themselves, and not knowing what the schools do teach, are liable to judge the faculties by the poorest specimen of all their graduates; and hence are honestly of the opinion that they can teach their pupils much better than they suppose they *can* be, or at most *are*, taught in the schools, and at a less rate of expense to the pupil, which is to most young men entering our specialty no small matter, as I can testify from extended observation. Now, instead of this being so, I hesitate not to say that there is not one practicing dentist on this continent who has the least claim to a first-class standing, who would not be much benefited by spending a session at any one of the dental colleges.

So soon as every dentist who has undertaken to help any young man into "the dentistry business" shall make it the prime requisition of his course of instruction that he shall attend two full courses of lectures on the subjects taught in medical and dental colleges, the last of which must be at a dental school, we shall have taken a step in the advance that not only he, but every member of the profession and the community shall have abundant cause to rejoice over, not only for the good it brings to the tutor and the pupil whom he thus assists to an honorable stand in the profession and community, but every one who shall enjoy him in his whole after-career.

Does any one ask how the tutor, who thus generously supports the dental schools by sending pupils in his influence to their halls, is to be benefited? I answer, upon the return of his pupil, he will of course get by sharp questioning the whole course, so far as it was understood, and thus partake of the concentrated wisdom of the faculty, so far as it had found lodgment in the pupil's mind, without himself becoming a matriculant at the expense of his false pride, time, and college fees. To *big*-little men, who desire to absorb all possible and give out no more than they must, this is ample inducement to send all their students, and thus keep pace with the progress of the most earnest and active minds in the whole land and profession in this country and abroad.

The low estimate at which dental faculties hold their services, and the lack of earnestness in the fulfillment of their function, is by no means a trivial cause of retardation to dental progress. So soon as each dental teacher in office or faculty shall take Paul's cue and "magnify mine office" to an approximate appreciation of its importance, we shall have swept away a deal of rubbish in the way not only of dental progress, but progress in a true physiology and pathology of the entire system. After which we shall as a body know how to distinguish between health and disease, and how and when to contribute so promptly to the well-being of the patients in our charge, that in a few generations we shall be blessed with a progression heart-cheering to every philanthropist that shall deal a death-blow to all our now most common operations of extracting and filling teeth, which in its turn shall annihilate the necessity for doses and doctors to an extent not dreamt of by the dull, plodding, willing-to-stay-in-past-attainment sort of profession and people.

Then laying aside all these dead weights to our progress, let us buckle on the armor of our high mission in the spirit of noble volunteers, who are willing to lose their own lives in the blessedness of saving those of others more dear to their intensified affections, and thus make it possible for us to enter into the wholeness of life commensurate with the brightest ideal of the goodness of use in the exercise of the functions of good and true men in individual and associated capacities of private and professional function in present and prospective life.

